# Footpad dermatitis and pain assessment in turkey poults using analgesia and objective gait analysis

**DOI:** 10.1080/00071668.2015.1077203

**Published:** 2015-11-16

**Authors:** C. Weber Wyneken, A. Sinclair, T. Veldkamp, L. J. Vinco, P. M. Hocking

**Affiliations:** ^a^ The Roslin Institute and Royal (Dick) School of Veterinary Studies, University of Edinburgh, Midlothian, UK; ^b^ Wageningen UR Livestock Research, Department Animal Nutrition, Wageningen, The Netherlands; ^c^ National Reference Centre for Animal Welfare, Istituto Zooprofilattico della Lombardia e dell’Emilia Romagna, Brescia, Italy

## Abstract

1. The relationships between litter moisture, footpad dermatitis (FPD) and pain in medium-heavy turkey strains was studied by gait analysis in two medium-heavy with and without analgesia (betamethasone or bupivacaine).
2. The relationship between FPD and litter moisture was linear above a breakpoint of 49% litter moisture, and there were no differences between the two breeds in susceptibility to FPD.
3. Gait analysis showed higher impulse, single support time, stride time and stance time in breed A compared to breed B. Significant interactions between breed, litter and analgesic for impulse, single support time and stride time were associated with higher means for breed A given saline injection on wet litter.
4. Data from betamethasone analgesia in Experiments 1 and 3 were combined for analysis. Peak vertical force was higher in saline- compared to betamethasone-treated birds. Compared to the wet (high FPD) litter treatments, birds on dry (low FPD) litter had greater speed and lower double support time and longer stride length. Turkeys kept on wet litter had a longer stride length compared to dry litter when given saline, whereas in betamethasone-treated birds the means were similar.
5. There were no differences between birds with or without bupivacaine analgesia. Peak vertical force was higher in breed A than B and in birds with a low FPD compared to a high FPD score.
6. It was concluded that breeds A and B did not differ in susceptibility to develop FPD when housed on wet litter but may have natural gait differences. Significant changes in gait parameters were associated with wet litter and with analgesic treatments. The results showed that FPD affected the gait of the turkeys and, combined with evidence of behavioural changes when given analgesia, suggest that footpad lesions are painful.

## INTRODUCTION

Footpad dermatitis (FPD) is a contact dermatitis of poultry associated with an immune response to wet litter (Mayne *et al*., 2007*a*) that affects the plantar aspect of the feet. It is a common condition in commercially grown turkeys and broiler chickens with a prevalence that varies between 20% and 41% of severe lesions (ulcers) and 78% to 59% of mild lesions (discoloration, erosion) (Ekstrand and Algers, 1997; Allain *et al*., 2013). Lesions commonly begin with skin discoloration, followed by inflammation, hyperkeratosis and erosions of the skin that may develop into ulcers and necrosis of the epidermis (Martland, 1984; Greene *et al*., 1985).

More recent research has shown that water alone is sufficient to develop FPD (Mayne *et al*., 2007*a*) and that the response to wet litter was linear above a certain minimum (Wu and Hocking, 2011). However, other factors such as diet, age and sex may affect the prevalence of FPD (Mayne, 2005) and there is anecdotal evidence that different commercial lines differ in their susceptibility to FPD. The objectives of the study were therefore first to obtain confirmatory evidence that the response to wet litter was linear above a specific minimum and second that two similar lines of turkeys differed in their susceptibility to FPD.

Gentle *et al*. (2001) showed that mechanothermal nociceptors are present in the scaly skin of chicken feet, and Martland (1984) suggested that FPD might be painful. Furthermore, turkeys with FPD induced by wet litter were less active and showed reduced behavioural complexity than birds on dry litter (Hocking and Wu, 2013). Differences in body weight and reluctance to place the feet on the ground in turkeys with FPD on wet litter might also indicate that lesions are painful (Mayne *et al*., 2007*a*). However, conclusive proof of pain requires more critical methods and the main purpose of this study was to assess the painfulness of FPD lesions by a combination of objective gait assessment in turkeys with and without lesions with and without analgesic intervention. Gait assessment was conducted on a tactile force and pressure measurement system that consists of plates of rubber-like material containing piezoelectric sensors to determine the force and pressure distribution exerted by each foot (Naas *et al*., 2010).

## MATERIALS AND METHODS

### Animals and husbandry

#### Experiment 1

A total of 240 male poults from breeder flocks of the same age (33 weeks) and from two medium-heavy commercial hybrids (A and B) were obtained from a commercial hatchery. The poults were housed on wood shavings in 24 pens containing 10 poults and from hatch to d 26 they were brooded under a heat lamp. The pens were 1.77 m × 1.25 m and were sealed with mastic at the junction of the pen wall and the floor to make them watertight. On arrival, poults were given water for 1 h followed by feed for 2 h and then 3 h darkness followed by 3 h light that was repeated until the onset of the planned dark period. Thereafter the birds were fed *ad libitum* on a commercial wheat and soya-based turkey starter feed presented in a feed trough; water was supplied in suspended bell drinkers and the photoperiod was 16 h light (07:30–23:30) and 8 h darkness per 24 h. Light intensities at d 1, 2 and from d 4 to the end of the experiment were 100, 50 and 12 lux, respectively. Ambient temperatures were 28^o^C, 26^o^C and 23^o^C, respectively, in weeks 1, 2 and 3–5. Relative humidity averaged 38% (range 31–53%) during the course of the experimental treatments. On d 27, the poults were reallocated in groups of 4 birds of one breed to 48 pens. The existing litter was removed and replaced with fresh wood shavings (4.6 kg) and the remaining birds were culled.

#### Experiment 2

Male poults (*n* = 60) of breed B were beak trimmed at the hatchery by an infrared method. They were housed at a rate of 10 or 11 per pen on white wood shavings and the feed, ambient temperature, photoperiod and light intensities were as described for Experiment 1, whereas water was supplied *ad libitum* in nipple drinkers (10 nipples in 80 cm, http://www.quillproductions.co.uk). Relative humidity averaged 42% (range 23–44%).

#### Experiment 3

A further 72 male poults of breed B were also beak trimmed at the commercial hatchery by an infrared method. Husbandry of the birds was as described for Experiment 2 except that the birds were fed on one of 4 diets based on maize or wheat as the main energy source and soya or non-soya ingredients as the main source of protein. The diets were fed as starter crumbs (0–4 weeks), rearer pellets (5–8 weeks) and grower pellets (9–12 weeks) based on commercial recommendations for diet composition at these three phases. Ambient temperatures were reduced from 23^o^C at 6 weeks to 20^o^C at 7 weeks, 18^o^C at 8 weeks and 16^o^C at 11 weeks of age. A total of 18 birds from each dietary treatment consisting of 6 from each of 3 pens were housed in a double pen (1.77 m × 1.25 m) at 64 d of age and were fed on the same diet that they had previously been given. Feed and water were provided *ad libitum* in a suspended tubular feeder and bell drinker. Relative humidity averaged 60% (range 45–75%).

### Treatments

#### Experiment 1

Experimental procedures were staggered over 2 days (24 pens on the described day and 24 on the next). Pens were randomly allocated to one of the following treatments: 1W1, 1W2 and 1W3, initially adding 1.0, 2.0 and 3.3 kg water/pen, respectively, in order to reach a target water content of approximately 30%, 40% and 50% and a dry (no water added) control, 1D. The same quantity of water was applied daily with a garden watering can during 7 d to the corresponding pens, starting from d 29. On the last day, after water was added and also 24 h later, litter samples were taken from all pens. Litter material was sampled at 30 cm from the 4 corners of each pen, mixed in a bucket and a sub-sample of approximately 100 g was placed in a disposable container and dried in a fan oven (Gallenkamp size one fan incubator) at 60^o^C for at least 7 d.

Two days after litter water addition started, a pair of birds from each pen was randomly selected to be injected into the breast muscle with either betamethasone (Betnesol Injection, RPH Pharmaceuticals AB, Haninge, Sweden) at a rate of 0.04 mg/kg or the same volume of saline (0.4 ml) for 5 consecutive days as described by Hocking *et al*. (2001). Treated birds were marked with an animal-safe spray on their back.

On d 37, 8 turkeys that had not been used for betamethasone and saline injections were randomly selected from each breed (4 with footpad score 0 and 4 with footpad score 6/7). Each bird was injected with 0.7 ml of either bupivacaine (3 mg/kg, Marcain, AstraZeneca, Luton, UK) or saline into the footpad using the dose recommended by Hocking *et al*. (1997). A delay of at least 15 min was observed for the bupivacaine to take effect before assessing gait on the Walkway, which was completed within one hour of the injection.

#### Experiment 2

On d 35, the turkeys were distributed to 12 pens of 5 birds per pen, and the litter was replaced with clean wood shavings. Each pen was randomly allocated to one of three water treatments (2D, 2W1 and 2W2), which started on d 38 with the litter water addition and continued for 7 consecutive days. The amount of water added daily was either 2.5 kg (2W1) or 4.0 kg (2W2) or half of this quantity in order to maintain the target moisture contents based on the litter scoring system of Tucker and Walker (1999) of score 2 (slightly damp) in 2W1 or score 3 (damp/tacky) in 2W2.

#### Experiment 3

Three different litter water treatments were allocated at random (3D, 3W1 and 3W2) within each diet from d 64 to d 79. The target moisture contents were 55% for 3W1 and 75% for 3W2. Litter was assessed to maintain a score of 3 (3W1, damp or tacky litter) or 5 (3W2, soggy, very wet and greasy litter, leaving a durable imprint when compressed or very slippery) as described by Tucker and Walker (1999).

On d 74, two birds from each pen were randomly selected and marked with an animal-safe spray and injected intramuscularly with either betamethasone (0.04 mg/kg) or saline for 6 consecutive days as described for Experiment 1.

### Experimental observations

#### Experiment 1

Turkeys were weighed on the first and last day of water addition, and water and food consumption for the experimental period (d 29–35) was also recorded. Footpad lesions were scored by one person on the last day of each experiment on an 8-point scale as described previously (Mayne *et al*., 2007*a*) and the highest score for both feet was recorded.

Gait analysis was conducted on d 36 on a tactile force and pressure measurement system (Walkway Research v. 7.02, Tekscan Inc., Boston, USA) using treated (betamethasone) and control (saline) turkeys from 1D and 1W3 pens (*n* = 48 birds). Prior to data collection, each turkey was weighed individually and given 2–5 min for habituation to the runway. The runway was 240 cm long x 60 cm high x 60 cm wide and contained the pressure platform (112 cm long × 59 cm wide) covered by a black rubber mat 5 mm thick at one end. A pen mate was placed at the other end of the walkway and the other bird was encouraged to walk through it as many times as necessary in order to get at least three good recordings (steady walking rhythm, with at least 4 steps on the sensor plates). Walkway data were collated in a laptop computer linked to the Walkway and gait parameters were subsequently extracted for analysis (Table 1).

**Table 1.** Definition of the gait parameters recorded on the Tekscan Walkway System (Walkway User Manual v.7.02x, pp. 208–213)

Variable	Definition
Speed (m/s)	Gait distance (from posterior heel of first stance to posterior heel of last stance) divided by gait time (time of first contact of first step to time of first contact of the last step registered on the sensor)
Peak vertical force (% of BW^1^)	The peak vertical force value reported as a percentage of the bird’s body weight
Impulse (% BW^1^/s)	The impulse value (force × time it acts over) reported as a percentage of the bird’s body weight impulse
Single support time (s)	The time the foot is in contact with the sensor, measured from the last contact of the opposing foot’s preceding stance to the first contact of the opposing foot’s next stance
Double support time (s)	Time over which the body is supported by both legs
Stance time (s)	The average time from first contact of the foot to last contact of the same foot
Stride length (cm)	Distance between the posterior heel points of two consecutive footprints of the foot in question (parallel to the line of progression)
Stride time (s)	Elapsed time between the first contacts of two consecutive footfalls of the foot in question

^1^BW, body weight. Peak vertical force is named maximum force in the user manual.

#### Experiment 2

Body weight, water and food consumption were recorded as in Experiment 1 for the experimental period (d 38–45). FPD and litter moisture were also determined as described for Experiment 1 but gait analysis was not conducted.

#### Experiment 3

The 12 pairs of birds in 6 randomly selected pens were weighed and gait analysis was conducted as described above on d 79 and the remaining birds on d 80. The procedures were the same as in Experiment 1 except that more runs were obtained to select 5 qualifying runs for assessment of gait parameters.

### Welfare inspection

All experiments and procedures were conducted after ethical approval under project licence number PPL60/45067. The health of the turkeys was inspected on a daily basis and severely affected birds were humanely killed. All turkeys were killed at the end of each experiment with an intravenous sodium pentobarbital overdose (Euthatal, Merial, Toulouse, France)

### Statistical analysis

Experiment 1 was a randomised block and Experiments 2 and 3 were completely randomised designs. Analysis of variance was conducted in GenStat (13th edition, http://www.vsni.co.uk/software/genstat). Treatment effects for the analyses of FPD score, body weight, feed and water intake were water treatment (and breed in Experiment 1). The mean values for litter moisture content and FPD scores from Experiments 1 and 2 were combined and analysed by a segmented, two-line regression model to fit linear and horizontal lines compared to quadratic and cubic polynomial models.

The model for the analysis of the gait parameters of both left and right feet included pen effects as blocks and litter and drug (with breed for Experiment 1) as treatment effects. The Walkway data from Experiments 1 and 3 for the betamethasone-treated birds were combined to increase the statistical power of the analysis. Breed in Experiment 1 was ignored and treatments 3W1 and 3W2 were combined. The block structure was pen nested within experiment and the treatment structure was litter (wet vs. dry) and drug (betamethasone or bupivacaine vs. saline). All data were checked for assumptions of normality and equality of variance by inspection of the residuals and residual variance plots. No data transformations were required.

## RESULTS

### Production variables

There were no statistically significant production differences between the two breeds in Experiment 1. Overall means (±SD) were 1690 ± 87.2 g for live weight at 4 weeks and 76 ± 5.6 g/d for live weight gain, 169 ± 2.6 g/d for feed intake and 321 ± 38.4 ml/d for water intake from 3 to 4 weeks of age. Corresponding means in Experiment 2 were 1866 ± 134.2 g, 82 ± 11.9 g/d, 131 ± 12.3 g/d and 304 ± 111.4 ml/d. Mean body weights and weight gains were similar across the three treatments in Experiment 3 and averaged 8506 ± 748 g and 233 ± 66 g/d, respectively. Averages of production traits and tests of significance for the treatments in all three experiments are provided in Supplemental Tables 1–3.

### FPD scores

Highly significant (*P < *0.001) differences occurred between treatments for litter moisture content in both Experiments 1 and 2, whereas FPD score was affected (*P < *0.001) only in Experiment 1. FPD score averaged over both breeds for 1D, 1W1, 1W2 and W3 were 0.8, 2.5, 4.4 and 6.2 (SED, standard error of a difference, 0.47, *P < *0.001) in Experiment 1 compared to 0.56, 0.94 and 0.38 (SED 0.567, not significant) for 2D, 2W1 and 2W2. Litter moisture (%) was 36, 59, 71 and 80 (SED 2.3, *P < *0.001), respectively, in Experiment 1 and 9, 30 and 43 (SED 3.70, *P < *0.001) in Experiment 2.

The segmented regression model resulted in a better fit than the quadratic polynomial (RSS = 1.40, *F*_3,8_ = 248.1 compared to RSS = 2.05, *F*_2,8_ = 95.7) in describing the relationship between FPD score and litter moisture (Figure) and the cubic term was not significant. The linear breakpoint on the *X*-axis for the two-line model was 48.9% ± 2.79% moisture (95% confidence interval 41.4%, 54.2%) with a slope of 0.174 ± 0.0197 FPD score/1% moisture. The breakpoint on the *Y*-axis was 0.71 ± 0.020 FPD score. Mean treatment FPD score plotted against mean litter moisture is presented in the figure with the fitted two-line regression.

**Figure.**
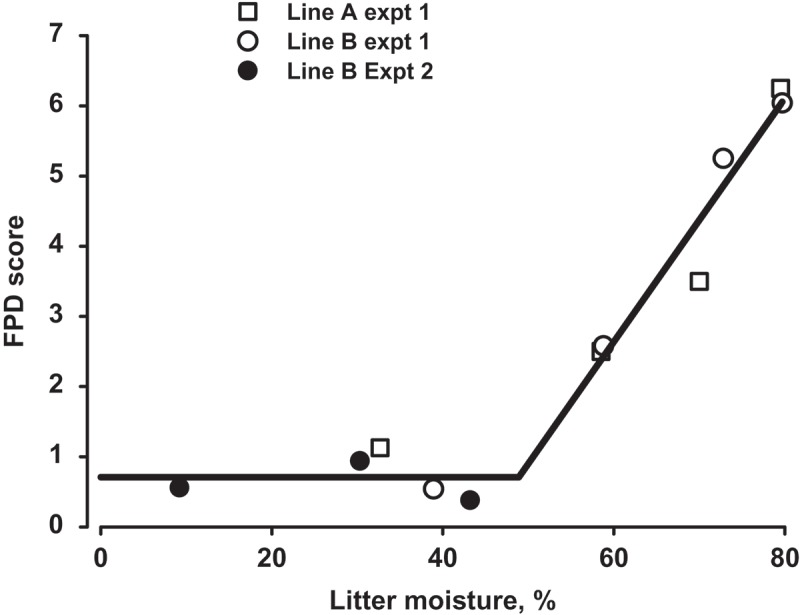
*Mean FPD score of different genetic lines of turkeys reared on different litter treatments plotted against mean litter moisture for Experiments 1 and 2. The fitted line is from a segmented regression model*.

Litter moisture for 3D, 3W1 and 3W2 treatments at the end of Experiment 3 averaged 36%, 59% and 63% (SED 2.9, *P < *0001) and mean FPD scores were 0.5, 6.1 and 6.3 (SED 0.43, *P < *0.001), respectively.

### Gait analysis using betamethasone analgesia

Gait analyses were conducted on the 1D and 1W3 treatments in Experiment 1. Data for two birds for the betamethasone treatment were not available, leaving *n* = 46 for analysis. The correlation between left and right feet was >0.8 for all variables except peak vertical force (*r* = 0.4) and the mean for both left and right feet was analysed.

Marginal means for breed, litter treatment and the use of analgesic are provided in Supplemental Table 4. There was a significant interaction (*P < *0.05) between breed, litter and analgesic for these same traits (impulse, single support time and stride time) that was associated with relatively high means for breed A on wet litter (high FDP) given saline injections (Supplemental Table 5). Averaged over litter and analgesic, breed A showed higher means than B for each of these variables (*P < *0.05).

In Experiment 3, one betamethasone-injected bird died before being tested. The marginal means for litter treatment and analgesia are presented in Supplemental Table 6. There was a significant interaction between litter and drug for stride length where the difference between birds with low and high FPD scores (dry vs. wet litter, respectively) was similar in betamethasone-treated birds and greater in birds given a saline injection. Mean stride length (cm) for 2D, 2W1 and 2W2 birds given betamethasone were 49.4, 51.2 and 48.3 compared to 51.8, 46.0 and 46.2 for saline-treated birds (SED 1.81 for comparisons on the same litter otherwise 1.71).

Marginal means for the main effects of the combined analysis from Experiments 1 and 3 are presented in Table 2. Peak vertical force was higher in saline- compared to betamethasone-treated birds (*F*_1,30_ = 7.25, *P = *0.012). Birds on dry (low FPD) compared to the wet (high FPD) litter treatments had greater speed (*F*_1,33_ = 4.68, *P = *0.038) and lower double support time (*F*_1,33_ = 4.81, *P = *0.035). A significant interaction between litter and drug occurred for stride length: there was a greater difference in saline-treated turkeys kept on wet compared to dry litter, whereas means were similar in betamethasone-treated birds on dry and wet litter. Means (cm) for birds on dry and wet litter, respectively, were 37.1 and 36.9 for betamethasone compared to 39.1 and 25.4 on the saline treatment (SED 1.00 within analgesic and 0.76 within litter).

**Table 2.** Marginal means and significance of main effects for the gait parameters in Experiments 1 and 3 combined for birds with low or high footpad scores and treated with daily intramuscular injections of analgesic (betamethasone) or saline

Main effect	Speed (m/s)	Peak vertical force (%BW^1^)	Impulse (%BW/s)	Single support time (s)	Double support time (s)	Stride length (cm)	Stride time (s)	Stance time (s)
Low FPD score	38.8	110.9	46.0	0.440	0.160	38.3	1.041	0.585
High FPD score	34.4	108.5	48.9	0.444	0.197	36.2	1.095	0.640
SED	2.05	1.90	2.047	0.0189	0.0116	0.836	0.0465	0.0250
Significance	0.038	NS	NS	NS	0.035	0.018	NS	0.035
Betamethasone	36.7	107.9	47.0	0.446	0.177	36.9	1.059	0.614
Saline	36.1	111.2	48.3	0.439	0.183	37.2	1.083	0.618
SED	1.43	1.26	1.546	0.0134	0.0084	0.54	0.0332	0.0221
Significance	NS	0.012	NS	NS	NS	NS	NS	NS

The means are for the average of left and right feet except for speed and double support time. ^1^BW, body weight; NS, not significant (*P *> 0.05).

### Gait assessment using bupivacaine analgesia

No statistically significant interactions between treatment effects were found when using bupivacaine. Marginal means for the main effects are presented in Table 3. Significant differences in peak vertical force were detected between breeds and litter (FPD score). Peak vertical force was higher in breed A than breed B and higher in birds with a low FPD score compared to a high FPD score. There were no differences between birds with or without analgesia.

**Table 3.** Marginal means and significance of main effects for the gait parameters in Experiment 1 for birds with low or high footpad scores and treated with a local injection of bupivacaine or saline

Main effect	Speed (m/s)	Peak vertical force (%BW^1^)	Impulse (%BW/s)	Single support time (s)	Double support time (s)	Stride length (cm)	Stride time (s)	Stance time (s)
Breed A	32.4	117.1	49.3	0.508	0.074	34.2	1.078	0.575
Breed B	30.6	111.1	48.3	0.492	0.095	32.8	1.093	0.583
Significance	NS	0.05	NS	NS	NS	NS	NS	NS
Low FPD	30.3	117.2	50.3	0.518	0.069	32.9	1.109	0.576
High FPD	32.7	111.0	47.4	0.482	0.100	34.1	1.062	0.581
Significance	NS	0.045	NS	NS	NS	NS	NS	NS
Bupivacaine	30.7	113.8	48.8	0.499	0.084	33.3	1.098	0.585
Saline	32.3	114.4	48.9	0.501	0.085	33.6	1.073	0.573
Significance	NS	NS	NS	NS	NS	NS	NS	NS
SED	2.11	2.73	2.20	0.0251	0.0193	1.443	0.048	0.0268

The means are for the average of left and right feet except for speed and double support time. ^1^BW, body weight; NS, not significant (*P *> 0.10).

## DISCUSSION

### Litter moisture and FPD

The present study confirms the previously reported linear relationship between FPD score and litter moisture above a certain minimum (Wu and Hocking, 2011). However, the minimum moisture content at which FPD began to develop was estimated to be 30% by Wu and Hocking (2011), whereas the breakpoint established in this experiment was higher (49% moisture). In both Experiments 1 and 2 in the present research, the relative humidity was very low, leading to comparatively rapid drying of the surface of the litter. Furthermore, the litter samples were taken from the full depth of litter and may not reflect the moisture content of the surface litter actually in contact with the feet. Taken together, the results emphasise the large number of environmental variables such as ambient temperature, ventilation rate, relative humidity and litter material that affect litter moisture content and the prevalence of FPD. It is important also to note that the FPD scores refer largely to the area of affected tissue which in general had not progressed to the stage of ulceration and significant loss of epidermal tissue.

### Breed and FPD

Breeds A and B are medium-heavy strains that are widely used in Europe and of similar performance characteristics. There were no differences between breeds in the first experiment, and the second hypothesis that the breeds used in this experiment differ in their susceptibility to FPD is rejected. Hocking and Wu (2013), using a similar system, found that lines of heavy turkeys developed FPD earlier than traditional lines, although the differences were not significant after 6 d. Conversely, they reported large differences between individuals within line which were not apparent in the present experiments. Nevertheless, because this is a model system, it is possible that a difference in breed susceptibility might exist in commercial situations.

### FPD, feed intake and weight gain

In Experiments 1 and 2, where feed intake and weight gain were recorded, no differences among litter treatments were found. Previous studies by Wu and Hocking (2011) showed that turkeys on wet litter had a higher feed intake but similar weight gain, whereas Mayne *et al*. (2007*a*) found lower body weights on wet litter, associated with a lower feed intake. The productivity of broiler chickens housed on wet litter was lower than in birds on dry litter (De Jong *et al*., 2014). It is possible that the low relative humidity affected these traits in the present experiments.

### Gait assessment

No major differences between treatments were observed when the FPD score on both feet were analysed and compared or the mean score was analysed and only the average is presented in the tables. In contrast to these results, Naas *et al*. (2010) found that peak vertical force differed between left and right legs in broiler chickens.

There is no published report of the Tekscan being used on turkeys, but in addition to the study on broiler chickens by Naas *et al*. (2010) there are reports of its use in cows and dogs (Lascelles *et al*., 2006; Shearer *et al*., 2013). The present research revealed differences between the two breeds in impulse, single support time, stride and stance time. It is possible that breeds A and B have naturally occurring gait differences with A taking longer in the different segments of the gait cycle compared to B. Whereas speed was not significantly different, it was numerically lower in breed B. Factors such as breed temperament might also explain these results, although, importantly, the interactions between litter, analgesia and litter treatments were one of scale rather than sign.

Most of the differences in gait parameters occurred between litter rather than drug treatments. Differences between breeds in Experiment 1 were inconsistent and the combined analysis of Experiments 1 and 3 was conducted to increase the power of the main experimental comparisons of litter (FPD score) and analgesic treatment. Wet litters led to higher footpad lesions, and gait differences are hypothesised to be due to birds experiencing pain or discomfort. In general, the results are consistent with this hypothesis. For example, walking speed was slower in birds that had been on wet litter: lame broiler chickens walked significantly slower than sound ones, and walking speed increased when an analgesic (carprofen) was administered (McGeown *et al*., 1999). Turkeys from wet litters spent a longer time supporting their body on both legs, which provides more stability (Caplen *et al*., 2013) and would be expected to increase with lameness. Stride length was shorter, reflecting shorter steps, and it has been shown that lame cows have a slower walking speed and perform shorter strides compared to sound animals (Flower *et al*., 2005). Finally, stance time was longer in turkeys kept on wet litter which may be interpreted as birds being more careful when placing a foot on the littered floor because of associated pain. Whereas birds with no footpad lesions kept on wet litter conditions might have altered gait in their home pen (see Sinclair *et al*., 2015), this is unlikely to have been carried over into the testing environment of the Walkway where the birds were evaluated under the same conditions. Nevertheless, animals with and without injuries must be observed under conditions that do not confound the behaviour of interest (Weary *et al*., 2006) and it will be important to confirm these results by conducting a similar study on naturally occurring lesions of different FDP scores in birds kept on the same litter.

Relatively few differences in the gait parameters were detected between saline- and betamethasone-treated turkeys. Peak vertical force showed a significant difference between breeds, consistent with the hypothesis of a natural gait difference, and between birds with low and high footpad lesions (Table 3). Peak vertical force was also higher in saline-injected birds (Table 2). Turkeys with high footpad scores may exert less force, therefore reducing stress on the musculoskeletal system produced during walking (Corr *et al*., 2007), a result which is consistent with that in lame broiler chickens reported by Naas *et al*. (2010) before and after (metanizole sodium) treatment.

Very few differences were detected between birds treated with bupivacaine and saline in Experiment 1, and this treatment was not repeated in Experiment 3: it is likely that the bupivacaine experiment was underpowered with too few birds being tested. Thus, peak vertical force was significantly higher in birds on dry litter (low FPD) scores compared to birds on wet litter (high FPD scores), a result that is inconsistent with the data from betamethasone treatment where there was no detectable difference.

Endogenous analgesia was first demonstrated in poultry by Hocking (1994) in broiler breeders with skeletal disease and by Gentle and Corr (1995) in an experimentally induced articular pain model in layer-type pullets. In addition, these authors showed that changes in motivation can supress pain by altering the attention of the animal away from pain (e.g. a novel environment). Thus, endogenous analgesia could explain the relative lack of differences between the analgesia and saline treatments in the Walkway environment if the bird was strongly motivated to reach its pen mate. Another possible explanation is that the footpad was not inflamed, preventing the analgesic from being effective, although this is inconsistent with the differences observed between the birds with and without FPD lesions (wet vs. dry litter) and previous research demonstrating an inflammatory response to wet litter in terms of both cellular and cytokine changes (Mayne *et al*., 2006, 2007*b*).

Betamethasone was effective in turkeys and broiler breeders with hip disorders (Duncan *et al*., 1991; Hocking, 1994), but to our knowledge, this drug has not been used for the assessment of pain associated with FPD in birds. A disadvantage of using betamethasone is the time required for the drug to achieve effective plasma concentrations and a suitable alternative non-steroid analgesic that did not possess this limitation would be advantageous in experiments to assess pain in birds with FPD. Betamethasone is also a potent glucocorticoid that may also affect behaviour (Liu *et al*., 2012; Berthon *et al*., 2014). Alternative analgesics that have been tested in turkeys include meloxicam and flunixin (Baert and De Backer, 2003) and the opioid butorphanol (Buchwalder and Huber-Eicher, 2005). The effectiveness of bupivacaine as an analgesic has been demonstrated in young layer chicks with experimentally induced articular pain (Hocking *et al*., 1997). Using a local analgesic had advantages when compared to betamethasone, in terms of involving less handling of the poults (only one injection, instead of repeated administration over several days). However, concerns existed due to the high volume injected into the footpad, especially with heavier birds. Hence, a more concentrated solution or a different drug that permitted a lower injection volume would be advantageous.

## CONCLUSIONS

The crucial role that water plays in the development of FPD is supported by the present research. However, there were no detectable differences in susceptibility to FPD in the two breeds that were tested in this experiment. The results showed that FPD affected the gait of the turkeys, which, together with further evidence of behavioural changes (Sinclair *et al*., 2015), is consistent with the hypothesis that footpad lesions are painful. Nevertheless, future experiments with improved analgesic intervention are necessary to obtain definitive evidence for pain and to assess the painfulness of different FPD scores. The Walkway was a useful tool that allowed objective gait assessment in turkeys to be made under experimental conditions, but birds may experience confounding motivations that compromise its use in some circumstances.

## DISCLOSURE STATEMENT

No potential conflict of interest was reported by the authors.

## Supplemental data

Supplemental data for this article can be accessed at http://dx.doi.org/10.1080/00071668.2015.1077203.

Supplementary Tables 1-6
